# Targeting non-coding RNAs and alarmins in myocardial ischemia-reperfusion injury: emerging molecular therapeutics for cardiothoracic surgery

**DOI:** 10.1186/s13019-026-04658-w

**Published:** 2026-07-29

**Authors:** Hussein Mussa Muafa

**Affiliations:** Department of Surgery, 21 September University for Medical and Applied Sciences, Sana’a, Yemen

**Keywords:** Myocardial ischemia-reperfusion injury, Non-coding RNA, microRNA, Long non-coding RNA, Circular RNA, HMGB1, Alarmins, Cardioplegia, Cardiothoracic surgery, RNA therapeutics

## Abstract

Myocardial ischemia-reperfusion injury (MIRI) remains a major unresolved cause of perioperative myocardial dysfunction after coronary artery bypass grafting, valve surgery requiring cardiopulmonary bypass, heart transplantation, and other procedures involving controlled ischemia followed by reperfusion. Reperfusion is essential for tissue salvage, yet it initiates mitochondrial reactive oxygen species generation, calcium overload, mitochondrial permeability transition pore opening, endothelial dysfunction, sterile inflammation, microvascular obstruction, arrhythmogenesis, and regulated cardiomyocyte death. This review provides an updated conceptual synthesis of the current framework of MIRI around two interacting molecular layers: non-coding RNAs (ncRNAs) and alarmins, also known as damage-associated molecular patterns. MicroRNAs, long non-coding RNAs, and circular RNAs regulate apoptosis, autophagy, pyroptosis, ferroptosis, angiogenesis, fibrosis, and macrophage polarization through post-transcriptional repression, competing endogenous RNA networks, and epigenetic scaffolding. In parallel, alarmins such as high-mobility group box 1 (HMGB1), S100 proteins, extracellular histones, mitochondrial DNA, and interleukin-33 link tissue injury to innate immune activation through Toll-like receptors, RAGE, inflammasome pathways, and ST2-dependent cardioprotective signaling. Mechanistic crosstalk, exemplified by the MALAT1/miR-200c/HMGB1 axis and the interaction between HMGB1 antagonism and miR-21-dependent survival signaling, provides a rationale for multi-target cardioprotection rather than single-pathway blockade. Translational progress is ongoing but remains in early stages. Although antisense oligonucleotides, small interfering RNAs, mRNA therapeutics, biomimetic nanoparticles, exosome-based delivery, and local perioperative administration through cardioplegia or epicardial injection provide potential translational routes, no ncRNA- or alarmin-directed therapy is currently established for routine prevention of surgical MIRI.

## Highlights

Myocardial reperfusion injury is driven by coordinated mitochondrial, inflammatory, proteostatic, endothelial and cell-death networks rather than one isolated pathway.

ncRNAs act as upstream network regulators of apoptosis, autophagy, ferroptosis, pyroptosis, fibrosis, angiogenesis and immune-cell polarization.

Alarmins such as HMGB1, S100A8/A9 and IL-33 translate sterile tissue injury into inflammatory or reparative signaling.

The MALAT1/miR-200c/HMGB1 axis illustrates how ncRNA dysregulation can amplify alarmin release and post-ischemic inflammation.

Cardiothoracic surgery offers a unique delivery window because cardioplegia and controlled reperfusion permit local, time-locked intervention.

## Introduction

Restoration of blood flow is the foundation of modern treatment for ischemic myocardium. In acute myocardial infarction, rapid reperfusion limits irreversible necrosis; in cardiac surgery, cardioplegia and controlled reperfusion allow complex operations while preserving myocardial viability. Yet reperfusion is biologically paradoxical. The return of oxygenated blood triggers a second wave of injury that may enlarge infarct size, impair contractile recovery, destabilize electrical conduction, and intensify postoperative low-cardiac-output states. The clinical consequences include myocardial stunning, no-reflow, ventricular arrhythmias, perioperative myocardial injury, graft or allograft dysfunction, and progressive adverse ventricular remodeling [1–4].

The pathobiology of MIRI is complex because ischemia and reperfusion disturb multiple systems at once. Ischemia causes ATP depletion, intracellular acidosis, lactate accumulation, sodium overload, calcium overload, and impairment of membrane pumps. Reperfusion then rapidly corrects pH, reintroduces oxygen, oxidizes accumulated succinate, and generates a burst of reactive oxygen species. These events promote mitochondrial permeability transition pore opening, cytochrome c release, activation of caspase-dependent and caspase-independent death pathways, endothelial swelling, leukocyte recruitment and microvascular obstruction [1–5].

Traditional cardioprotective approaches have focused on single targets such as calcium handling, free-radical scavenging, ischemic conditioning, mitochondrial pore inhibition or anti-inflammatory therapy. Despite compelling preclinical data, translation has often been disappointing, partly because MIRI is not a single biochemical event but a network disease. This creates a strong rationale for therapeutics that modulate upstream regulatory nodes. Non-coding RNAs and alarmins are especially relevant because they sit at the interface between intracellular stress sensing, innate immunity, proteostasis, cell death, fibrosis and vascular repair [6–10].

**Fig. 1:**
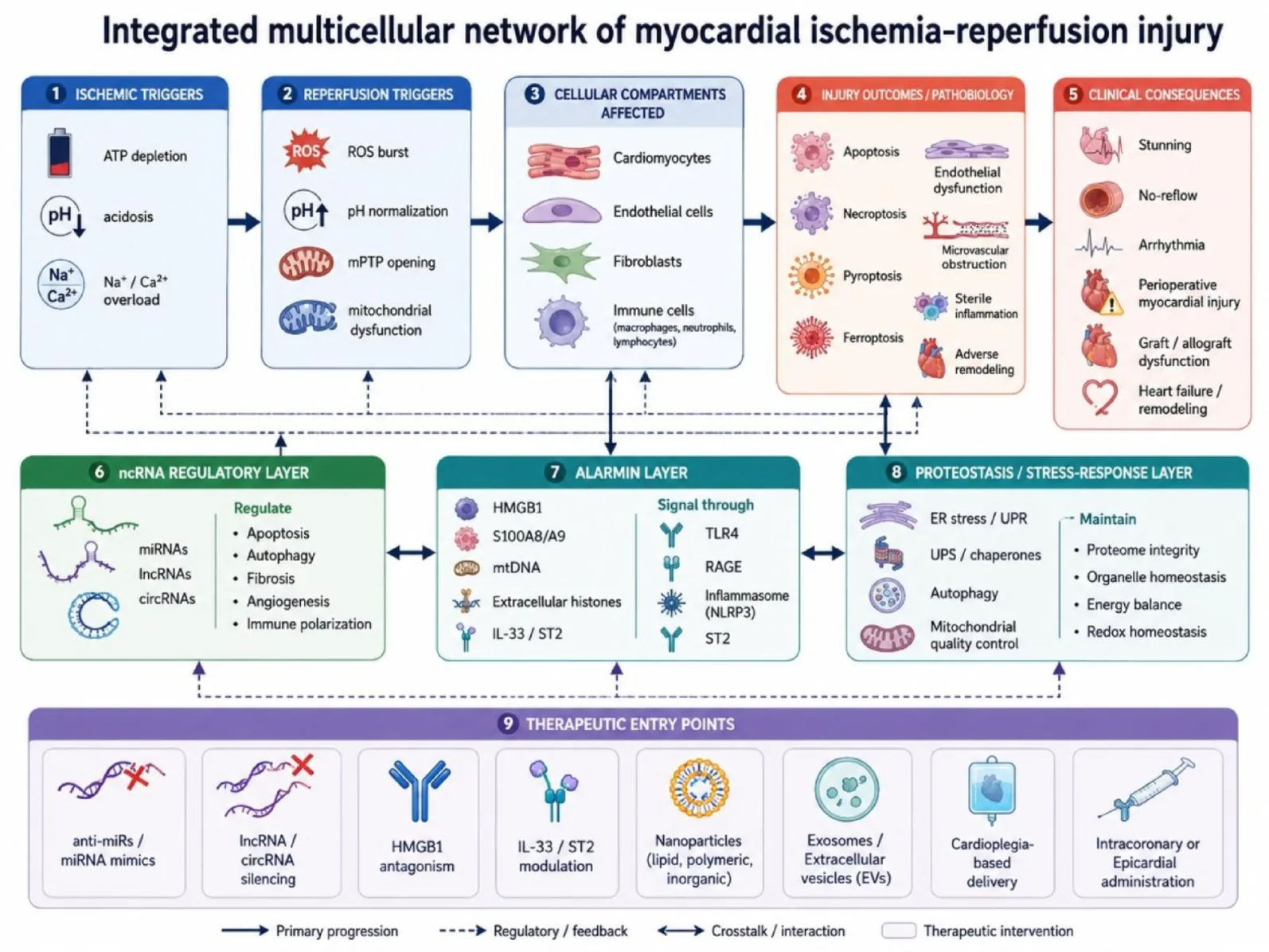
Integrated molecular network of myocardial ischemia-reperfusion injury. MIRI results from convergent ischemic metabolic stress, reperfusion-induced oxidative injury, regulated cell death, innate immune activation, proteostasis disruption and ncRNA-mediated epigenetic control

This translational gap is supported by several representative human trials [11–16]. In acute ST-elevation myocardial infarction, cyclosporine-mediated mitochondrial permeability transition pore inhibition in the CIRCUS trial did not improve clinical outcomes or prevent adverse left ventricular remodelling despite strong experimental rationale [11]. Complement inhibition with pexelizumab in APEX-AMI also failed to reduce mortality in patients undergoing primary PCI [12]. Similarly, TRO40303 in the MITOCARE study and intravenous sodium nitrite in the NIAMI trial did not significantly reduce infarct size when administered before reperfusion [13, 14]. In the surgical setting, large trials of remote ischemic preconditioning, including ERICCA and RIPHeart, did not improve major clinical outcomes after cardiac surgery [15, 16]. These examples indicate that failure of translation is not simply due to lack of promising targets, but reflects inadequate myocardial delivery, redundancy of injury pathways, variable comorbid phenotypes, anaesthetic and operative confounders, and reliance on surrogate endpoints that may not predict clinically meaningful benefit. The integrated injury network and proposed intervention points are summarized in Fig. 1.

This review synthesizes the field from a cardiothoracic surgical perspective. It synthesizes mechanistic pathways, clinically relevant ncRNA and alarmin targets, emerging delivery technologies, human trial signals, and pragmatic steps needed to convert molecular cardioprotection into perioperative practice.

### Literature Selection and Search Strategy

To ensure transparency, a structured literature search was performed across databases including PubMed, Web of Science, and Scopus, covering a time window from January 2005 to May 2026. The search strategy utilized keywords including (“Non-coding RNA” OR “MicroRNA” OR “lncRNA” OR “circRNA” OR “Alarmins” OR “HMGB1”) AND (“Myocardial Ischemia Reperfusion” OR “Cardiothoracic Surgery”). Inclusion criteria emphasized peer-reviewed original research, systematic reviews, and major clinical trials focusing on mechanistic interplay and therapeutic interventions, while isolated case reports were generally excluded.

## Pathophysiological foundation of MIRI

### Ischemic phase: energetic collapse and ionic instability

During ischemia, interruption of oxygen delivery suppresses mitochondrial oxidative phosphorylation and forces cardiomyocytes to rely on anaerobic glycolysis. ATP depletion reduces activity of ATP-dependent ion pumps, while lactate and proton accumulation lower intracellular pH. To compensate, the sodium-hydrogen exchanger extrudes protons at the cost of intracellular sodium accumulation. The altered sodium gradient then reverses or impairs the sodium-calcium exchanger, producing cytosolic and mitochondrial calcium overload. Sarcoplasmic reticulum dysfunction adds further calcium instability, setting the stage for hypercontracture, mitochondrial swelling and activation of calcium-dependent proteases during reperfusion [1, 2].

### Reperfusion phase: ROS burst and mitochondrial permeability transition

The early minutes of reperfusion are dominated by abrupt biochemical shifts. Reintroduction of oxygen allows rapid oxidation of ischemia-accumulated succinate and electron leakage through respiratory-chain complexes. Reactive oxygen species damage lipids, proteins and nucleic acids and amplify mitochondrial dysfunction. Together with calcium overload and rapid pH normalization, oxidative stress promotes opening of the mitochondrial permeability transition pore. mPTP opening collapses mitochondrial membrane potential, halts ATP generation, and releases pro-death signals. This is a decisive event linking reversible ischemic stress to irreversible cardiomyocyte loss [1, 3, 5].

### Microvascular injury and sterile inflammation

MIRI is not limited to cardiomyocytes. Endothelial cells, pericytes, resident macrophages, neutrophils, platelets and complement all contribute to the no-reflow phenomenon. Endothelial swelling, glycocalyx disruption, vasoconstrictor imbalance, neutrophil plugging, platelet microthrombi and capillary leakage produce microvascular obstruction even after epicardial blood flow has been restored. Necrotic and severely stressed cells release alarmins that activate pattern-recognition receptors, resulting in cytokine generation, inflammasome activation and further leukocyte recruitment [6, 17, 18].

### Integration of ncRNAs and Alarmins within the Broader Pathophysiological Network

It is critical to note that while this manuscript emphasizes ncRNAs and alarmins, they do not dominate MIRI biology in isolation; rather, they represent two important components of a much broader pathophysiological network. They must be fully integrated with other established determinants of MIRI, such as mitochondrial metabolism—specifically the ischemic accumulation of succinate and Complex I reactivation driving extensive reactive oxygen species (ROS) production. Furthermore, this network intimately involves immunometabolism, the formation of neutrophil extracellular traps (NETs), complement activation, and the stability of the endothelial glycocalyx. Additionally, mitochondrial dynamics and mitophagy interact continuously with ncRNA regulators to dictate cell survival or death, emphasizing that MIRI is a highly integrated, multi-system failure rather than a pathway-specific disease.

## Proteostasis and autophagy: the degradation-repair axis

Protein quality control determines whether stressed cardiomyocytes recover or progress to death. Ischemia and reperfusion damage proteins through oxidation, calcium-dependent cleavage, misfolding and aggregation. The endoplasmic reticulum unfolded protein response, ubiquitin-proteasome system, molecular chaperones, mitochondrial proteases and autophagy cooperate to restore proteostasis. The same systems may become maladaptive when excessively activated or when stress exceeds their capacity [19–22].

Autophagy is a central example of this duality. Moderate autophagy during ischemia can preserve viability by removing damaged mitochondria and aggregated proteins. During reperfusion, however, ROS-driven overactivation may degrade essential cellular components and contribute to autophagic cell death. ncRNAs regulate this balance through targets such as Beclin-1, ATG5, ATG7, ATG12, LC3, p62, SIRT1 and PI3K/Akt/mTOR. This makes autophagy a therapeutic zone rather than a simple on/off target [7, 23, 24].

## Non-coding RNAs as master regulators of MIRI

### MicroRNAs

MicroRNAs are short non-coding RNAs, generally 18–24 nucleotides long, that repress gene expression by binding target mRNAs. A single microRNA can regulate many transcripts and a single transcript can be regulated by several microRNAs. This many-to-many biology is highly relevant to MIRI, where injury results from integrated apoptotic, inflammatory, vascular and fibrotic programs rather than one gene product [8–10, 25].

Several microRNAs aggravate injury. miR-1 is enriched in muscle and is released during acute myocardial injury; it has been linked to apoptosis, arrhythmogenic remodeling and adverse prognosis. miR-155 promotes inflammatory macrophage activation and NF-kB-dependent signaling. miR-92a suppresses angiogenic repair and its inhibition derepresses vascular growth pathways. miR-25 impairs calcium handling through SERCA2a regulation. miR-132 is a central mediator of hypertrophy and fibrosis, making it a major target for anti-miR therapy in post-infarction remodeling [26–31].

Other microRNAs are protective in specific contexts. miR-21 enhances survival signaling by suppressing PTEN/PDCD4-related pathways, although its role may differ between cardiomyocytes, fibroblasts and immune cells. miR-133 and miR-499 are associated with anti-apoptotic and anti-hypertrophic programs. The translational challenge is therefore not simply to increase or inhibit a named microRNA, but to define the correct cell type, disease phase, dose, timing and delivery vehicle [26, 32].

### Long non-coding RNAs

Long non-coding RNAs exceed 200 nucleotides and regulate gene expression through chromatin remodeling, transcriptional scaffolding, RNA-protein interactions, and competing endogenous RNA behavior. In MIRI, lncRNAs can sponge protective microRNAs, modulate mitochondrial function, alter autophagic flux, and coordinate inflammatory and fibrotic phenotypes [8, 33].

MALAT1 is among the most studied pathogenic lncRNAs in MIRI. It is induced by hypoxia/reoxygenation and can amplify apoptosis, oxidative stress and inflammation through several microRNA-dependent axes, including interactions with HMGB1-related signaling. Other lncRNAs such as MIAT, CHRF, NRF and Mirt1 have been implicated in inflammatory recruitment, autophagy, necrosis or fibrosis. In contrast, H19, HOTAIR, SNHG1 and selected context-specific lncRNAs may reduce apoptosis, pyroptosis or oxidative injury by restoring regulatory microRNA networks [33–37].

### Circular RNAs

Circular RNAs are covalently closed transcripts generated by back-splicing. Their resistance to exonuclease degradation makes them stable regulators and attractive circulating biomarkers. Many circRNAs function as microRNA sponges. circNCX1, derived from the sodium-calcium exchanger gene, promotes apoptosis by sponging miR-133a-3p and derepressing CDIP1. circHIPK3 and circTLK1 have been associated with autophagy and TNF-related apoptotic signaling, whereas circPAN3 and circZNF292 may activate protective pathways depending on model and timing [38–40].

Recent studies have expanded the circRNA landscape in MIRI beyond early candidates such as circNCX1, circHIPK3 and circTLK1. circMIRIAF has been reported to aggravate myocardial ischemia-reperfusion injury by acting through the miR-544/WDR12 axis, thereby linking circRNA dysregulation to oxidative stress and cardiomyocyte injury [41]. Inhibition of circDGKZ has been shown to attenuate MIRI by targeting the miR-345-5p/TLR4 pathway, suggesting a direct connection between circRNA activity and innate immune receptor signalling [42]. circDhx32, identified in mouse cardiac I/R and hypoxia-reoxygenation models, promotes inflammatory responses by binding FOXO1 and suppressing AdipoR1-dependent AMPK/NF-kB signalling; cardiomyocyte-specific circDhx32 knockdown improved cardiac function, reduced infarct size and decreased inflammatory cytokine release [43]. Additional emerging circRNAs, including circRNA_0007444 and circANKIB1, have been linked to cardiomyocyte inflammation, apoptosis or ferroptosis through miR-16-5p/MFN2 and miR-452-5p/SLC7A11-related mechanisms [44, 45]. These findings show that circRNAs are not merely passive biomarkers but may operate as miRNA sponges, RNA-binding protein scaffolds, epitranscriptomic targets and inflammatory amplifiers during reperfusion. However, most evidence remains preclinical; therefore, claims regarding circRNA-targeted therapy should be framed as hypothesis-generating until validated in human myocardial tissue and perioperative cohorts.Major miRNA, lncRNA and circRNA candidates relevant to MIRI and perioperative translation are summarized in Table 1.

**Table 1:** Major non-coding RNA candidates relevant to myocardial ischemia-reperfusion injury and perioperative translation

Molecule	Class	Predominant reported effect	Key mechanisms/targets	Translational relevance
miR-1	miRNA	Mostly pathogenic	Targets anti-apoptotic and electrophysiological pathways; associated with cardiomyocyte injury and arrhythmia	Potential biomarker; inhibition requires caution because of muscle specificity
miR-21	miRNA	Context-dependent/protective in cardiomyocytes	PTEN/Akt and PDCD4-related survival pathways; interacts with HMGB1 inhibition	Candidate mimic or endogenous mediator of alarmin-targeted cardioprotection
miR-92a	miRNA	Anti-angiogenic/pathogenic after MI	Suppresses pro-angiogenic endothelial programs	Anti-miR-92a has human proof-of-mechanism in angiogenesis-related settings
miR-132	miRNA	Pathogenic remodeling signal	Hypertrophy and fibrosis gene networks	CDR132L has entered randomized cardiovascular trials; post-MI HF-REVERT was safe but neutral on primary remodeling endpoint
MALAT1	lncRNA	Pathogenic in many models	Sponges protective miRNAs; derepresses HMGB1 and inflammatory pathways	Silencing may attenuate inflammation, apoptosis and electrical instability
H19	lncRNA	Often protective/context-dependent	Regulates autophagy and microRNA networks such as miR-143/ATG7	Potential target for restoring adaptive autophagy
LIPCAR	lncRNA	Biomarker	Mitochondria-derived circulating lncRNA associated with remodeling	Risk stratification after myocardial injury
circNCX1	circRNA	Pathogenic	Sponges miR-133a-3p and derepresses CDIP1	Potential anti-circRNA strategy; biomarker candidate
circPAN3	circRNA	Protective in selected models	Modulates autophagy and survival pathways	Potential mimic or diagnostic target

## Alarmins in myocardial reperfusion injury

Alarmins, or damage-associated molecular patterns, are endogenous molecules released or exposed during necrosis, severe stress or extracellular matrix disruption. Intracellularly, they may have normal structural or regulatory functions; extracellularly, they signal danger through receptors such as TLR2, TLR4, RAGE, NOD-like receptors and ST2. In sterile myocardial injury, this danger signaling substitutes for pathogen recognition and drives an immune response that is initially reparative but can become destructive [6, 17, 18].

### HMGB1

High-mobility group box 1 is a nuclear chromatin-binding protein that stabilizes nucleosomes and participates in DNA repair and transcriptional regulation. After necrosis or active immune-cell secretion, extracellular HMGB1 binds TLR4 and RAGE, activates NF-kB and MAPK pathways, and induces cytokines such as IL-1beta, IL-6 and TNF-alpha. In MIRI, early HMGB1 release can enlarge injury through inflammation, endothelial activation and apoptosis; later HMGB1 activity may contribute to repair, angiogenesis and remodeling. This biphasic biology means that timing of inhibition is essential [46–49].

### S100 proteins, mitochondrial DNA and extracellular histones

The S100 family includes calcium-binding proteins with divergent cardiovascular roles. S100A8/A9, also called calprotectin, is linked to neutrophil activation, plaque vulnerability and adverse cardiovascular events. S100A12 can signal through RAGE and contribute to vascular inflammation. In contrast, S100A1 supports calcium handling and contractile recovery in experimental ischemia. Other alarmins, including extracellular mitochondrial DNA and histones, can activate TLR9, cGAS-STING, complement and inflammasome pathways, intensifying sterile inflammation and microvascular injury [50–54].

### IL-33/ST2 signaling

Interleukin-33 is an IL-1 family alarmin with important cardioprotective features. It signals through the membrane ST2 receptor and may suppress HMGB1, IL-6 and TNF-alpha expression, reduce apoptosis through p38 MAPK-related mechanisms, and promote angiogenesis through AKT/eNOS signaling. Soluble ST2, which acts as a decoy receptor, is clinically associated with cardiac stress and adverse outcomes. The IL-33/ST2 axis therefore represents both a protective pathway and a biomarker-linked system [55, 56]. The principal alarmins involved in MIRI, their receptor pathways, dominant biological effects and therapeutic interpretation are summarized in Table 2.

**Table 2:** Principal alarmins implicated in myocardial ischemia-reperfusion injury and their therapeutic interpretation

Alarmin	Principal receptors/signaling	Dominant effect in MIRI	Therapeutic interpretation
HMGB1	TLR4, RAGE, NF-kB, MAPK	Early injury amplification; possible late repair	Acute, time-limited antagonism may be beneficial; prolonged blockade may impair repair
S100A8/A9	RAGE, TLR4	Neutrophil-rich inflammation, oxidative stress and plaque vulnerability	Biomarker and potential anti-inflammatory target
S100A1	Intracellular calcium-handling targets	Improves contractile reserve in experimental models	Gene or protein-based augmentation is conceptually cardioprotective
Mitochondrial DNA	TLR9, cGAS-STING, inflammasome pathways	Links mitochondrial injury to innate immune activation	Strategies that preserve mitochondrial integrity may reduce mtDNA release
Extracellular histones	TLRs, complement and endothelial injury pathways	Cytotoxic endothelial and microvascular injury	Neutralization is promising but remains preclinical in MIRI
IL-33	ST2L; p38 MAPK; AKT/eNOS	Anti-apoptotic and pro-angiogenic in selected settings	Potential cardioprotective pathway; soluble ST2 complicates signaling

## Crosstalk between ncRNAs and alarmins

The strongest rationale for targeting ncRNAs and alarmins together is their bidirectional crosstalk. ncRNAs regulate alarmin expression, release and receptor signaling; alarmins in turn reshape ncRNA expression in cardiomyocytes, endothelial cells, fibroblasts and immune cells. This creates self-amplifying loops in which hypoxic stress induces an lncRNA, the lncRNA suppresses a protective microRNA, the loss of microRNA repression increases HMGB1 or another inflammatory mediator, and the resulting cytokine burst further modifies ncRNA expression [34, 46].

The MALAT1/miR-200c/HMGB1 axis is a representative pathway. MALAT1 upregulation during hypoxia/reoxygenation sequesters miR-200c, which normally represses HMGB1. HMGB1 derepression activates TLR4/RAGE signaling, amplifies oxidative stress and cytokine generation, and contributes to cardiomyocyte apoptosis and electrical instability. Experimentally, MALAT1 knockdown can reduce inflammation and restore more stable electrophysiological behavior [34].

A second clinically interesting interaction is the link between HMGB1 antagonism and miR-21. HMGB1 Box A or related HMGB1-blocking strategies may reduce inflammatory signaling, but part of the downstream cardioprotection appears to require miR-21-mediated activation of survival pathways. This suggests that successful perioperative cardioprotection may require simultaneous suppression of danger signaling and activation of endogenous survival networks rather than indiscriminate anti-inflammatory therapy [31, 47].

**Fig. 2:**
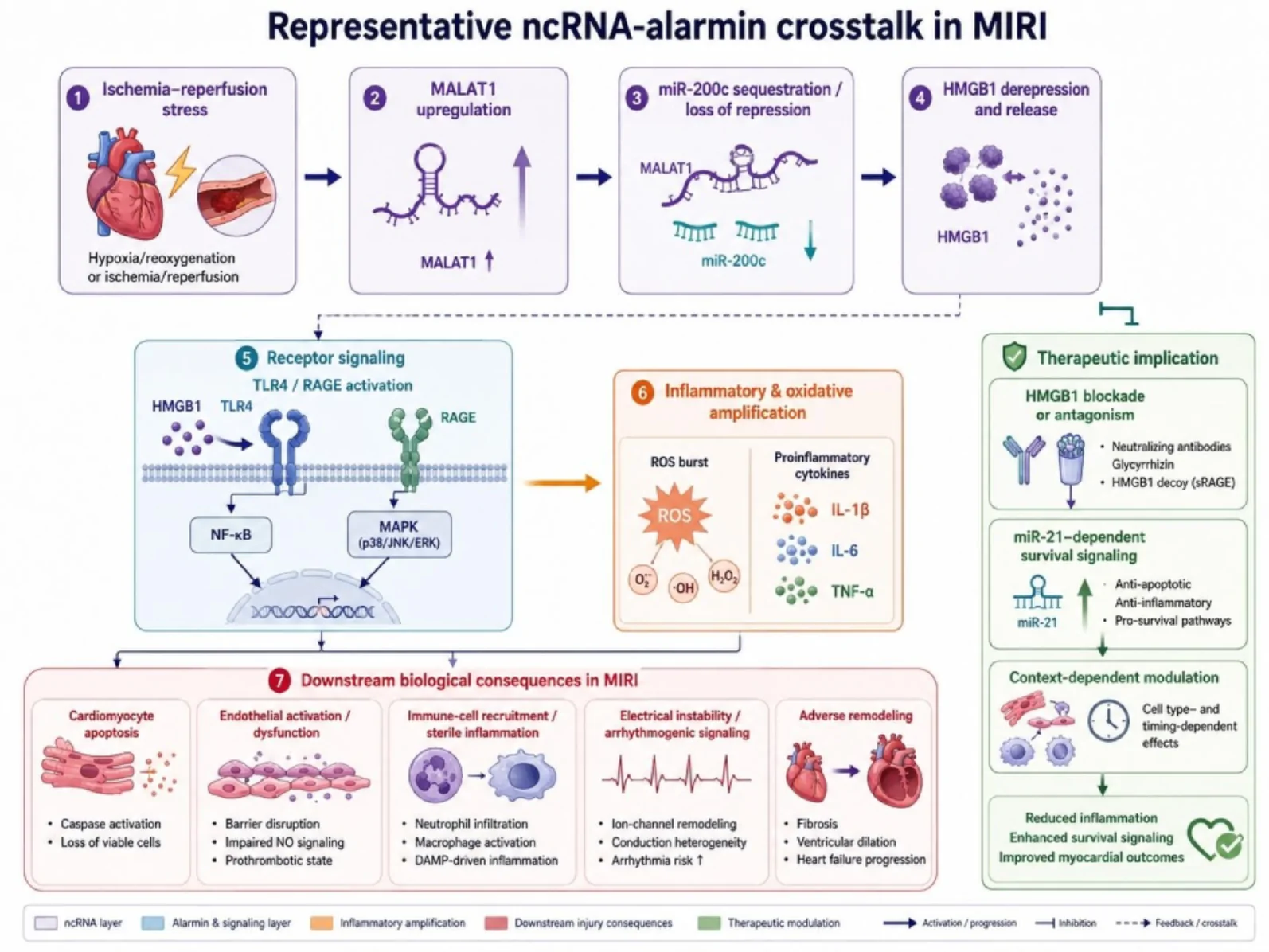
Representative ncRNA-alarmin crosstalk. MALAT1 can sequester miR-200c, derepress HMGB1, and amplify TLR4/RAGE-dependent inflammation, oxidative stress and cardiomyocyte injury

### Cell-type-specific actions of shared ncRNA-alarmin mediators

A key translational issue is that the same ncRNA or alarmin may exert divergent effects depending on the target cell type and timing after reperfusion. In cardiomyocytes, miR-21 is often protective by suppressing pro-apoptotic pathways such as PTEN/Akt, PDCD4 and FasL-related signalling, whereas in fibroblasts it may support extracellular matrix deposition and profibrotic remodelling through TGF-beta/SMAD and ERK-MAPK-related pathways. In endothelial cells, miR-21 and miR-92a can influence nitric oxide bioavailability, endothelial survival, angiogenesis and microvascular repair; therefore, systemic inhibition or replacement may have opposing myocardial and vascular effects. In immune cells, miR-21, miR-155 and related miRNAs shape macrophage polarization, neutrophil recruitment and cytokine generation, which can either amplify early injury or support later resolution depending on context.

HMGB1 provides a similar example of cell-type and phase dependence. Cardiomyocyte necrosis passively releases HMGB1 as a danger signal, endothelial HMGB1/RAGE/TLR4 activation contributes to barrier dysfunction and leukocyte adhesion, fibroblast exposure may influence scar formation and matrix remodelling, and macrophage-derived HMGB1 can amplify NF-kB-dependent IL-1beta, IL-6 and TNF-alpha release. Conversely, delayed HMGB1 signalling may participate in angiogenesis and tissue repair. IL-33/ST2 signalling also differs across compartments: membrane ST2 signalling may protect cardiomyocytes and support reparative immunity, whereas soluble ST2 can act as a decoy receptor and is associated clinically with myocardial stress and adverse outcomes. These examples argue against broad systemic modulation and support time-limited, compartment-aware delivery strategies. This representative ncRNA-alarmin amplification loop is illustrated in Fig. 2.

## Delivery strategies for RNA- and alarmin-directed cardioprotection

The therapeutic promise of ncRNA modulation depends on delivery. Naked RNA is vulnerable to nuclease degradation, renal clearance, poor membrane permeability and off-target distribution. Chemical modification, lipid or polymeric encapsulation, conjugation, local injection and biomimetic carriers are therefore central to translation [57–60].

### Lipid, polymeric and biomimetic nanoparticles

Lipid nanoparticles have transformed systemic RNA delivery, but myocardial targeting remains challenging. Polymeric systems such as PLGA, chitosan and polyethyleneimine derivatives can protect cargo and allow controlled release. Biomimetic nanoparticles coated with platelet, neutrophil, macrophage or erythrocyte membranes may improve immune evasion and target injured endothelium or inflamed myocardium. Nanozymes that mimic superoxide dismutase or catalase can neutralize ROS bursts and may be combined with RNA payloads in future multi-modal systems [57, 60].

### Exosome and extracellular vesicle platforms

The discussion of extracellular vesicle (EV)-based delivery in MIRI must be expanded beyond MSC-derived exosomes. Cardioprotective EVs have also been extensively investigated from alternative sources, prominently including plasma-derived exosomes which protect the myocardium from ischemia-reperfusion injury [61–62]. Furthermore, other critical cell sources include cardiac progenitor cells (CPCs) [63–64] and endothelial cells [65]. Because EV cargo, targeting properties, and biological effects are strongly influenced by the parental cell source, comparing these platforms is essential. For instance, CPCs and endothelial cell-derived EVs may possess inherent homing properties or specific microvascular reparative cargo, rendering them highly relevant for translating targeted therapies in myocardial ischemia-reperfusion injury. Major barriers include manufacturing reproducibility, cargo quantification, biodistribution, sterility, and regulatory classification [66, 67].

### Cardioplegic, intracoronary and epicardial delivery

**Fig. 3:**
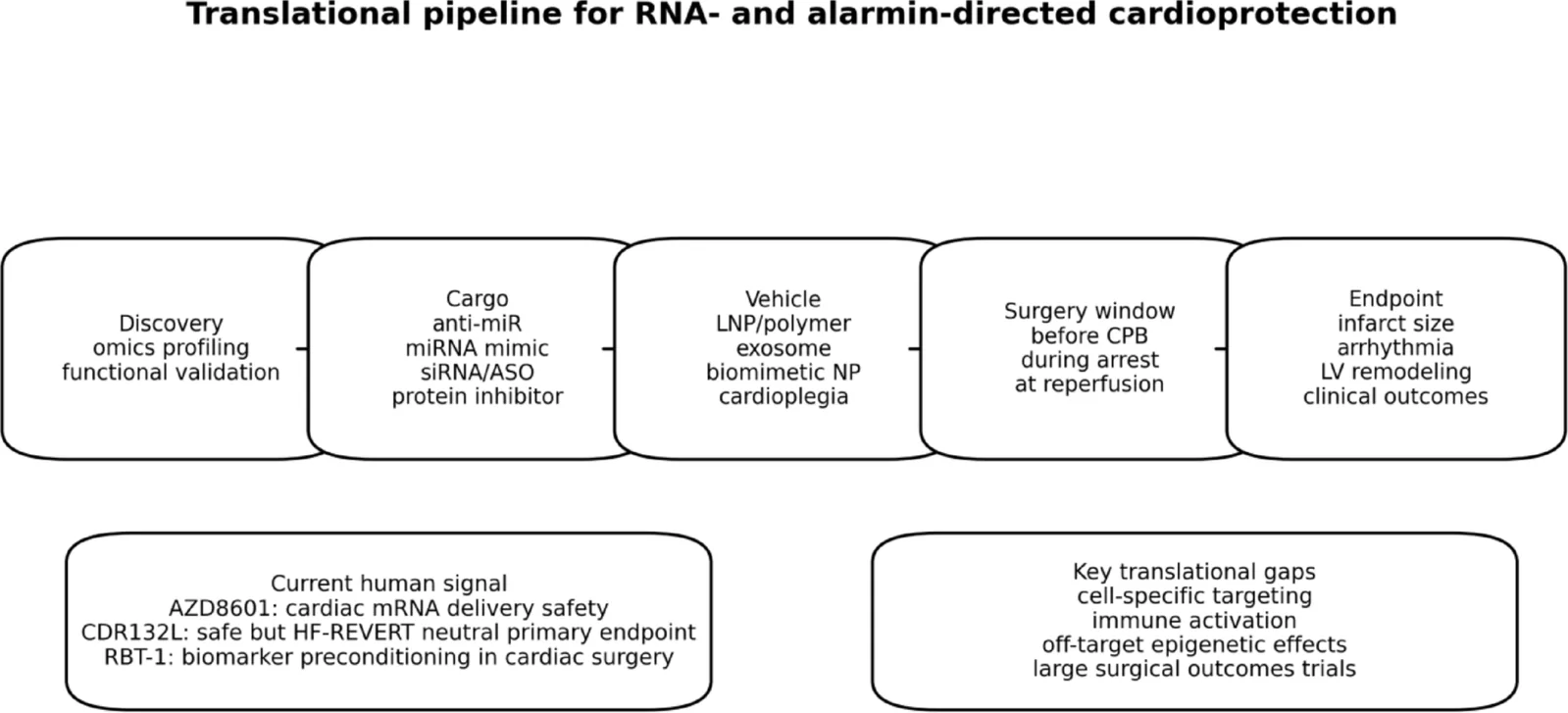
Translational pipeline for RNA- and alarmin-directed cardioprotection. Candidate discovery must be linked to cargo selection, delivery route, surgical timing and clinically meaningful endpoints

Cardiothoracic surgery provides a uniquely controllable therapeutic window. Unlike spontaneous myocardial infarction, the timing of ischemia and reperfusion during CABG, valve surgery and transplantation is known in advance. Therapeutics can be administered before aortic cross-clamping, mixed into cardioplegia, delivered into the aortic root, infused intracoronarily at reperfusion, or injected epicardially into ischemic territories. This local strategy may achieve higher myocardial exposure with less systemic toxicity, but the evidence should be interpreted as feasibility-supporting rather than definitive superiority. The EPICCURE experience with VEGF-A mRNA injected directly into the myocardium during CABG supports the practical feasibility and short-term safety of perioperative cardiac mRNA delivery, although it was not designed to prove protection against MIRI as a primary clinical endpoint [68, 69]. Preclinical studies also support the biological plausibility of local or intracoronary extracellular vesicle delivery: MSC-derived exosomes administered around reperfusion reduced infarct size, attenuated inflammatory cell infiltration and shifted macrophages toward a reparative phenotype in experimental MIRI [66, 70], while engineered exosome-based RNA delivery has shown target engagement in ischemic myocardium [71]. Therefore, cardioplegic, intracoronary and epicardial delivery routes should be described as promising and surgically practical approaches that require formal comparison of myocardial retention, biodistribution, compatibility with cardioplegia, CPB-related degradation, immune activation and dose-related toxicity before clinical adoption [69, 72]. The proposed translational pipeline connecting candidate discovery, cargo selection, delivery route, surgical timing and clinical endpoints is illustrated in Fig. 3. The major delivery platforms, candidate cargoes, potential advantages and key limitations for perioperative RNA- and alarmin-directed cardioprotection are summarized in Table 3.

**Table 3:** Delivery platforms and perioperative administration routes for RNA- and alarmin-directed cardioprotection

Platform	Cargo examples	Advantages	Key limitations
Lipid nanoparticles	siRNA, mRNA, anti-miR	Clinically validated RNA platform; tunable formulation	Liver tropism, complement activation, limited cardiomyocyte specificity
Polymeric nanoparticles	miRNA mimics, antagomiRs, antioxidant drugs	Controlled release and cargo protection	Endosomal escape and toxicity depend on chemistry
Biomimetic nanoparticles	ROS scavengers, mPTP inhibitors, RNA cargo	Injury homing and immune evasion via membrane coating	Complex manufacturing and characterization
Exosomes/EVs	miR-21, miR-133, anti-inflammatory cargo	Low immunogenicity and natural cell communication	Batch variability, scalability and regulatory uncertainty
Cardioplegic delivery	anti-miR, siRNA, alarmin inhibitor	Local high-concentration exposure during predictable ischemia	Needs compatibility with cardioplegia, temperature and surgical workflow
Epicardial injection	mRNA, plasmid, hydrogel/RNA systems	Precise surgical placement	Regional rather than global distribution

## Clinical translation and current evidence

Clinical translation of these delivery systems remains highly speculative. While this review discusses numerous delivery vehicles, almost none currently have convincing efficacy data in human trials for MIRI. The transition to clinical practice is significantly hindered by challenges in standardizing isolation protocols, batch-to-batch cargo variation, and the strict requirement for tissue-selective delivery to avoid systemic clearance. These strategies remain experimental, and clinical readiness is not yet established beyond current preclinical evidence [57, 68, 69].

CDR132L, an antisense inhibitor of miR-132, illustrates both progress and caution. Early-phase studies supported safety and biological target engagement, and HF-REVERT tested the concept in patients with reduced left ventricular ejection fraction after myocardial infarction. Recent reported data indicate that CDR132L was well tolerated but did not significantly improve the primary endpoint of left ventricular end-systolic volume index compared with placebo in the overall post-MI population. This does not invalidate miR-132 biology, but it emphasizes that patient enrichment, timing, endpoint selection and disease stage are critical for RNA therapeutics [29, 30, 72–74].

MRG-110, an anti-miR-92a strategy, demonstrated proof-of-mechanism for derepressing angiogenic pathways in human tissue-repair settings [75]. Its relevance to MIRI lies in the central role of microvascular repair and border-zone angiogenesis, although definitive cardiac outcome data are still needed. AZD8601, an mRNA encoding VEGF-A, demonstrated safety and tolerability after direct myocardial injection during CABG, supporting the surgical feasibility of local mRNA delivery [68, 69]. RBT-1, a stannic protoporphyrin and iron sucrose combination, produced a biomarker-defined preconditioning response in cardiac surgery patients, illustrating a parallel non-RNA strategy for cytoprotective priming before predictable ischemia [76, 77]. Selected human translational signals and clinically relevant interventions are summarized in Table 4.

**Table 4:** Current human translational signals for RNA- and alarmin-directed cardioprotection

Intervention/trial	Target or concept	Population/setting	Current interpretation for cardiothoracic translation
CDR132L / HF-REVERT	Anti-miR-132; reverse remodeling	Reduced LVEF after MI	Safe but neutral primary endpoint in overall post-MI population; may guide enrichment strategies
CDR132L ongoing remodeling studies	Anti-miR-132 in chronic HF phenotypes	HF with LV hypertrophy or reduced/mildly reduced EF	Tests whether stage and phenotype influence benefit
MRG-110	Anti-miR-92a; angiogenesis	Human tissue-repair proof-of-mechanism	Potential relevance to border-zone angiogenesis; cardiac outcomes unproven
AZD8601 / EPICCURE	VEGF-A mRNA	CABG patients with impaired systolic function	Demonstrated feasibility and safety of local myocardial mRNA delivery
RBT-1 / NCT04564833	Preconditioning via HO-1 and cytoprotective biomarkers	CABG/valve surgery requiring CPB	Supports perioperative cytoprotective priming; not an ncRNA agent
HMGB1 antagonists	Box A, glycyrrhizin, antibodies	Mostly preclinical MIRI	Promising but timing and repair-phase effects must be defined

## Proposed perioperative research framework

A publishable translational program in cardiothoracic surgery should not jump directly from promising animal data to broad clinical use. A staged pathway is more defensible. First, candidate ncRNAs or alarmins should be validated in human myocardial tissue, coronary sinus blood, plasma and perioperative samples collected across the ischemia-reperfusion timeline. Second, mechanistic models should confirm causality in cardiomyocytes, endothelial cells, fibroblasts and macrophages. Third, delivery compatibility with cardioplegia, hypothermia, heparinization, protamine, cardiopulmonary bypass circuits and common anesthetic drugs should be tested. Fourth, early trials should use mechanistic endpoints such as troponin kinetics, coronary sinus cytokines, cardiac MRI edema/infarct markers, strain imaging and arrhythmia burden. Finally, larger studies should evaluate clinically meaningful outcomes such as low-cardiac-output syndrome, inotrope requirement, atrial and ventricular arrhythmias, acute kidney injury, ICU length of stay, heart-failure hospitalization and mortality. A staged perioperative research framework linking discovery, mechanistic validation, delivery testing, early clinical trials and outcomes trials is presented in Table 5.

Patient selection is likely to determine success. High-risk groups include patients with reduced preoperative ejection fraction, diabetes mellitus, chronic kidney disease, older age, prolonged cross-clamp time, redo surgery, left main or multivessel coronary disease, heart transplantation, and anticipated ischemic durations. Molecular enrichment using baseline or early reperfusion ncRNA/alarmin profiles may identify patients with exaggerated inflammatory or remodeling phenotypes who are more likely to benefit.

**Table 5:** Proposed staged perioperative research framework for ncRNA- and alarmin-directed cardioprotection

Stage	Main aim	Recommended methods	Decision gate
Discovery	Identify robust human targets	Perioperative plasma/coronary sinus RNA-seq, proteomics, alarmin panels	Target reproducible across cohorts and associated with injury severity
Mechanistic validation	Prove causality	Human iPSC-cardiomyocytes, endothelial co-culture, macrophage systems, animal MIRI	Target modulation improves survival or vascular function
Delivery testing	Optimize myocardial exposure	Cardioplegia compatibility, nanoparticle biodistribution, EV cargo assays	Adequate myocardial retention without toxicity
Early clinical trial	Demonstrate safety and biological effect	Randomized dose-escalation; troponin, cytokines, MRI/echo strain, rhythm monitoring	Safety plus target engagement
Outcomes trial	Prove clinical value	Multicenter cardiac-surgery RCT	Reduction in perioperative myocardial injury or major postoperative complications

## Safety, regulatory and ethical considerations

RNA and alarmin therapies raise specific safety issues. Anti-miRs and siRNAs can affect unintended transcripts, alter immune signaling, or persist longer than expected. Delivery vehicles may trigger complement activation, thrombocytopenia, hepatic uptake or inflammatory reactions. Alarmin blockade could suppress necessary repair or host-defense functions, particularly after transplantation or in patients with infection risk. These concerns are amplified in cardiac surgery, where patients are exposed to anticoagulation, extracorporeal circuits, hypothermia, ischemia, transfusion and postoperative immune dysregulation.

Regulatory development should therefore include biodistribution studies, immunogenicity testing, interaction with standard cardioplegia, dose-reversibility assessment, long-term monitoring for arrhythmias and fibrosis, and explicit stopping rules. Ethical recruitment should emphasize that most ncRNA-alarmin strategies are investigational and that biomarker improvement does not automatically equal clinical benefit.

## Limitations of current evidence

Several limitations restrict the field. Although more human trials have been recently added to the literature, it must be explicitly emphasized that the vast majority of the mechanistic discussion and evidence presented herein rely almost exclusively on rodent models, in vitro hypoxia/reoxygenation studies, and cultured cardiomyocytes. These models do not fully replicate the complexity of human cardiothoracic pathophysiology.

Furthermore, the field is frequently challenged by the irreproducibility of ncRNA findings and conflicting biomarker studies. This inconsistency is largely attributable to variations in RNA quantification sensitivity due to their low concentrations in biofluids, a lack of standardized extraction protocols, and model heterogeneity. Different animal strains exhibit conflicting baseline inflammatory responses and mortality rates. The influence of anesthesia and surgical trauma in these models can also significantly confound cardiovascular physiology, yielding divergent results. Cell-specific effects are often underdefined; a microRNA that protects cardiomyocytes may promote fibrosis in fibroblasts or alter macrophage behavior.

## Conclusion

MIRI is a network-level injury process in which mitochondrial dysfunction, proteostasis disruption, regulated cell death, microvascular injury and sterile inflammation interact dynamically. ncRNAs and alarmins are not peripheral markers of this process; they are upstream regulators and amplifiers that can determine whether reperfused myocardium recovers, remodels or fails. The most promising future strategies will combine precise RNA modulation, time-limited alarmin targeting, and delivery systems tailored to the surgical window. Cardioplegia-based or epicardial delivery may give cardiothoracic surgery a unique advantage over systemic therapy for spontaneous infarction. However, successful translation will require rigorous human validation, phenotype-guided patient selection, careful safety monitoring, and randomized trials powered for clinically meaningful perioperative outcomes.

At present, ncRNA- and alarmin-directed interventions should not be presented as ready for routine perioperative use. Their value will depend on whether future studies can demonstrate reproducible human target engagement, safe myocardial delivery and improvement in clinically meaningful outcomes beyond biomarker reduction.

## Data Availability

No datasets were generated or analysed during the current study.
